# A Near-Deterministic Mutational Hotspot in *Pseudomonas fluorescens* Is Constructed by Multiple Interacting Genomic Features

**DOI:** 10.1093/molbev/msac132

**Published:** 2022-06-16

**Authors:** M J Shepherd, J S Horton, T B Taylor

**Affiliations:** Milner Centre for Evolution, Department of Biology & Biochemistry, University of Bath, Claverton Down, Bath BA2 7AY, United Kingdom; Milner Centre for Evolution, Department of Biology & Biochemistry, University of Bath, Claverton Down, Bath BA2 7AY, United Kingdom; Milner Centre for Evolution, Department of Biology & Biochemistry, University of Bath, Claverton Down, Bath BA2 7AY, United Kingdom

**Keywords:** mutation hotspot, Mutation bias, predicting evolution

## Abstract

Mutation—whilst stochastic—is frequently biased toward certain loci. When combined with selection, this results in highly repeatable and predictable evolutionary outcomes. Immotile variants of the bacterium *Pseudomonas fluorescens* (SBW25) possess a “mutational hotspot” that facilitates repeated occurrences of an identical de novo single nucleotide polymorphism when re-evolving motility, where ≥95% independent lines fix the mutation *ntrB* A289C. Identifying hotspots of similar potency in other genes and genomic backgrounds would prove valuable for predictive evolutionary models but to do so we must understand the genomic features that enable such a hotspot to form. Here, we reveal that genomic location, local nucleotide sequence, gene strandedness, and presence of mismatch repair proteins operate in combination to facilitate the formation of this mutational hotspot. Our study therefore provides a framework for utilizing genomic features to predict and identify hotspot positions capable of enforcing near-deterministic evolution.

A growing body of evidence has revealed that mutation bias is a key determinant of evolutionary outcomes (Couce and Tenaillon 2019; Cano et al. 2022; Monroe et al. 2022). Contrary to the expectation that mutations simply provide a random supply of genetic diversity for selection to act upon, differences in mutation rates across genomic position (Monroe et al. 2022) and mutation types (Cano et al. 2022) can introduce bias into the mutational spectra that has been shown to shape adaptive trajectories. In some circumstances, such biases can be strong enough that a particular position mutates at a rate far higher than expected by chance, generating what is referred to as a mutational hotspot (Zhang et al. 2018; Horton et al. 2021). Mutation bias is of key interest to those wishing to make forecasts of adaptive evolution, as mutational hotspots can facilitate highly repeatable, and by extension reliably predictable, adaptive outcomes. Identifying such hotspots across the bacterial domain and understanding their role in adaptive evolution is therefore an important challenge.

In previous work, we found that a mutational hotspot drove repeatable outcomes in immotile variants of *Pseudomonas fluorescens* SBW25 (denoted AR2; see Alsohim et al. 2014; Taylor et al. 2015). During the re-evolution of flagellar motility, >95% of replicate lines realized an identical de novo single nucleotide polymorphism (SNP), A289C in the *ntrB* gene (Horton et al. 2021). Just six synonymous SNPs defined whether strong mutation bias occurred at position 289 within the *ntrB* locus, and these changes were theorized to impact the formation of a single stranded DNA secondary structure (Horton et al. 2021). Elucidating biased hotspots of similar potency in other bacterial genomes would inform predictive evolutionary models and bring us closer to forecasting adaptive trajectories. But, we must first understand the generalizable genomic principles that allow such hotspots to occur.

In this work, we use the *P. fluorescens* mutational hotspot as a model system to elucidate the key genomic features responsible for facilitating heavily biased localized mutation. As this hotspot is sufficiently potent to generate an identical mutation in ≥95% of instances, the system provides a unique setting where a hotspot can endure to some degree when partially disassembled. This allows us to augment genomic features known to affect mutation bias in isolation and, in combination, and quantify their individual and combinatorial impact on the mutational hotspot.

We began with the hypothesis that the *ntrB* 289 hotspot is reliant on the formation of single-stranded DNA hairpins. If true, then a slew of genomic features can interfere with or facilitate this hotspot-generating mechanism (Trinh and Sinden 1991; Viswanathan et al. 2000; Hoede et al. 2006; Wang and Vasquez 2017). Hairpin formation does not generate mutation on its own, but rather indirectly by way of its interference of DNA polymerase, where it can force the replisome to stall (Voineagu et al. 2008). DNA polymerase fidelity is influenced by replication timing and its correlated genomic position (Dillon et al. 2018), with replisomes being less vulnerable to stalling closer to the origin of replication (OriC). Therefore the hairpin’s effect may be weakened when replication fidelity is higher. We investigated this hypothesis by translocating the locus to a genomic position ∼348 kbp closer to OriC.

Translocation was accomplished by knockout of the native *ntrBC* operon, followed by re-introducing *ntrBC* on a miniTn7 transposon insertion system that reliably inserts in a single defined chromosomal site downstream of the *glmS* gene (Choi and Schweizer 2006; fig. 1*A*). This conserves native operon structure, regulatory elements, and strandedness (fig. 1*B*). Translocating the *ntrBC* operon did not significantly impact expression of either gene according to RT-qPCR expression analysis (supplementary table S1, Supplementary Material online). This method measures a population aggregate of expression profiles and therefore we cannot dismiss differences between evolving subpopulations; however, the assay does provide confidence that subsequently observed changes in mutation spectra are due to genomic context rather than gene expression. The intact hotspot in its native location (∼376 kbp from OriC) leads to 95.2% of motility rescuing mutations being the *ntrB* SNP A289C, and 100% of replicate lines evolving within 6 weeks (fig. 2). The *ntrBC* translocation strain (AR2 miniTn7[*ntrBC*-Lag], ∼28 kbp from OriC) displayed a nonsignificant but increased median time to emergence of motility compared to AR2 from 3.83 to 5.35 days (*P* = 0.035, Dunn test), accompanied by a reduction in the rate of adaptation, with 95.65% lines evolving in 6 weeks (fig. 2*A*).

**Fig. 1.**
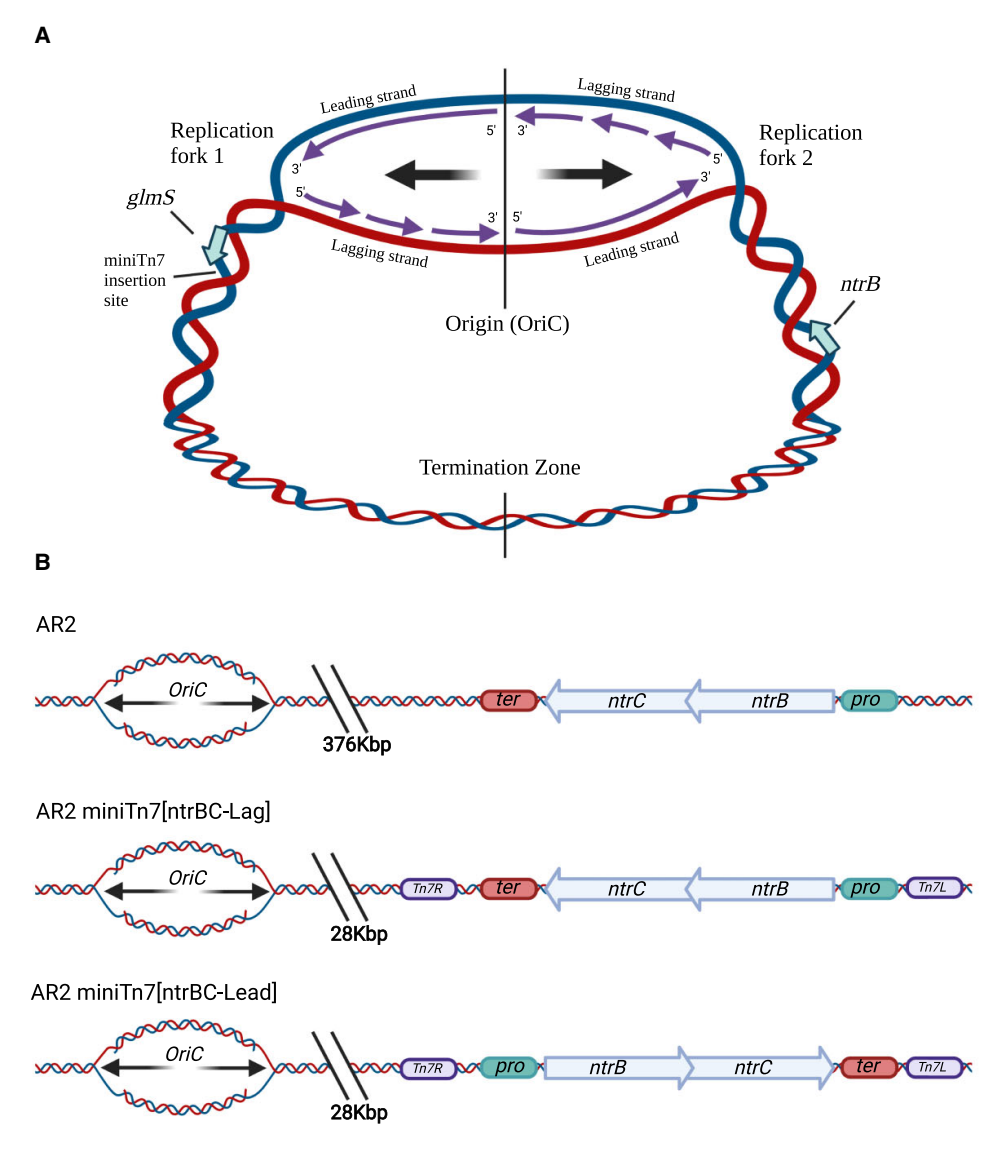
Manipulation of chromosomal locus and DNA strandedness of *ntrBC*. Large arrows indicate direction replication fork movement. (*A*) Bisymmetrical structure of the circular bacterial chromosome undergoing theta-replication, with two mirror-image replication forks moving out from the OriC. Synthesis of leading and lagging strands is shown by smaller arrows within each replication fork. (*B*) Altered genetic contexts of the *ntrBC* locus. Orientation with respect to the replication fork and distance from OriC show for ancestral (AR2) and the engineered strains AR2 miniTn7[*ntrBC*-Lag] and AR2 miniTn7[*ntrBC*-Lead]. Pro and ter denote *ntrBC* promoter and terminator regions respectively. Tn7R and Tn7L denote miniTn7 transposon flanking sites.

**Fig. 2.**
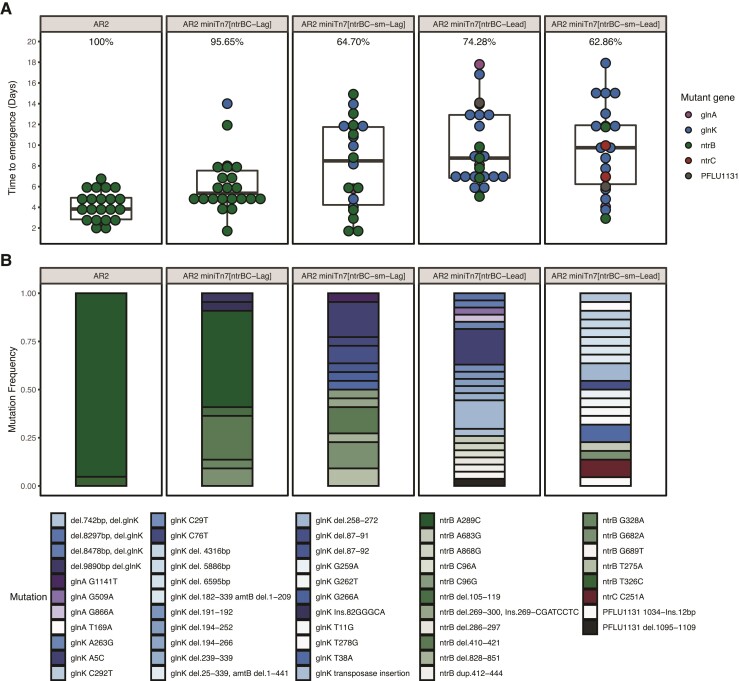
Impact of *ntrBC* translocation, gene strandedness and synonymous variation on mutation bias for rescuing flagellar motility in AR2-based strains. (*N* for each condition (evolved/total): AR2 – 21/21, miniTn7[*ntrBC*-Lag] – 22/23, miniTn7[*ntrBC*-sm-Lag] – 22/34, miniTn7[*ntrBC*-Lead] – 26/35, miniTn7[*ntrBC*-sm-Lead] – 22/35). (*A*) Time to emergence of motility in days for each *ntrBC* strain background. Boxplots display mean and quartile values. Datapoints for individual replicate lines are shown and colored by mutant gene identified. The percentage of replicates evolving motility within 6 weeks is given above each boxplot. (*B*) Frequency of de novo mutations identified in motile isolates. Each horizontal bar is a unique mutation represented by unique colors. Mutations in the same gene are grouped with shades of the same colour (*ntrB* = greens, *glnK* = blues, *glnA* = purples, PFLU1131 = grays, *ntrC* = red).

Translocating *ntrBC* resulted in decreased potency of the A289C hotspot (fig. 2*B*). Alternative de novo *ntrB* mutations occurred at raised frequencies in AR2 miniTn7[*ntrBC*-Lag], including a 12 bp deletion Δ410–421 (22.7%) and the SNP G682A (9.1%). Aside from *ntrB* mutations, 9.1% of mutations were observed in *glnK*, a gene encoding an NtrB-repressor (Hervás et al. 2009) that has also been observed to permit *ntrBC*-mediated rescue of motility (Taylor et al. 2015). These mutations came at the expense of mutation *ntrB* A289C in the translocated lines. The frequency of the SNP dropped from 95.2% in AR2 to 50% in AR2 miniTn7[*ntrBC*-Lag], yielding a significantly different mutation spectrum overall (*P* = 0.0015, Chi-square test). Furthermore, local nucleotide context continued to play a major role in mutation frequency at this site, as the introduction of six synonymous changes around position 289 in the novel genomic context (closer to OriC) generated a similar effect to that observed previously (Horton et al. 2021), with A289C frequency falling from 50% to 0% (fig. 2*B*).

These results show that genomic position closer to OriC lowers hotspot potency but does not remove it entirely, until however subsequent synonymous changes are introduced. This supports the broader hypothesis that replisome stalling at a hairpin site facilitates heavily biased mutation at *ntrB* 289. It should be noted, however, that these observations may be explained by alternative interacting mechanisms. DNA polymerase fidelity is dependent on both replication timing (Dillon et al. 2018), as well as local nucleotide context including nucleotide triplets (Long et al. 2014) and possibly flanking dinucleotides (Krawczak et al. 1998). Therefore, the two mechanisms may additively facilitate the hotspot without requiring the formation of a hairpin. Similarly, the rate of stalling may differ between the two genomic locations due to differences in local DNA topology (Postow et al. 2001), rather than by replication timing.

Stalled replisomes are vulnerable to generating mutations in part because they are vulnerable to collisions with RNA polymerases (Paul et al. 2013). Head-on collisions occur when genes are encoded on the lagging replicative strand, meaning that the two complexes process DNA in opposing directions, resulting in head-on contact. As *ntrB* is natively encoded on the lagging strand, head-on collisions may increase mutation frequency across the locus. However, if a DNA hairpin is stalling the replisome, then collisions with RNA polymerase may be enriched to occur at the hotspot site (Wang and Vasquez 2017), driving the localized mutation bias. Alternatively, a mechanism independent of collisions may still be strand dependent, as replisome-stalling hairpin structures have more opportunity to form when encoded on the lagging strand (Bikard et al. 2010). Therefore, if hairpin-replisome interactions or collisions are essential for mutagenesis, swapping the strand encoded by the gene should remove the hotspot, even when the local nucleotide sequence that facilitates replisome stalling remains intact. We experimentally examined the effect of gene strandedness by manipulating the encoded position of the *ntrBC* genes from the lagging to the leading DNA strand at the new genomic position (fig. 1*B*).

We first observed that switching the strandedness of the *ntrBC* locus on the miniTn7 transposon (AR2 miniTn7[*ntrBC*-Lead]) impacted the rate of adaptation, increasing median time to emergence relative to AR2 miniTn7[*ntrBC*-Lag] from 5.35 to 8.75 days (*P* = 0.0038, Dunn test) (fig. 2*A*). The percentage of replicate populations evolving within 6 weeks also decreased from 95.65% for AR2 miniTn7[*ntrBC*-Lag] to 74.26% for AR2 miniTn7[*ntrBC*-Lead]. The switch from lagging to leading strand additionally eradicated the mutational hotspot effect. The *ntrB* A289C SNP was no longer observed in the mutational spectra, and other de novo *ntrB* mutations accounted for only 22.2% of motility rescuing mutations. Mutations were instead observed in other previously identified motility-granting mutational targets *glnK, glnA*, and PFLU1131 at frequencies of 66.7%, 7.4%, and 3.7%, respectively (fig. 2*B*), producing a vastly different mutation spectrum to its lagging strand counterpart (*P* = 0.00005, Chi-square test). Loss of the *ntrB* A289C hotspot mutation could not be explained by a drop in viability or motility fitness for this SNP in the altered genomic contexts, as no significant difference in motility speed (*P* = 0.2667, Dunn test) was found when this SNP was engineered on the leading or lagging strand (supplementary fig. S1, Supplementary Material online).

The mutational data therefore show that gene strandedness is essential to hotspot formation. Additionally, if replisome stalling followed by RNA polymerase collisions are driving hotspot mutagenesis (see supplementary table S2, Supplementary Material online), encoding the gene on the leading strand would nullify head-on contact. As such, synonymous mutations that prevent stalling, possibly by abolishing hairpin formation or creating less mutable local sequence, should no longer impact the observed mutational spectrum, as head-on collisions are removed in either case. Neither AR2 miniTn7[*ntrBC*-Lead] nor AR2 miniTn7[*ntrBC*-sm-Lead] realized any *ntrB* A289C mutations. Alternative mutations within the locus dropped from 22.2% to 9.1% following synonymous mutation; however, *ntrB* mutations seen in the AR2 miniTn7[*ntrBC*-sm-Lead] background were A683G and G682A, which were also seen in other strains in this study. The rest of the mutations for AR2 miniTn7[*ntrBC*-sm-Lead] were 72.7% *glnK*, including multiple observations of Δ258–272 also observed in the non-sm dataset, 4.5% PFLU1131, and 9.09% *ntrC*. Overall, there was no significant difference in the mutational target on the locus level (*P* = 0.51, Chi-square test). And despite novel mutational routes being discovered, there was also no significant difference in observed mutation spectra for individual mutations between the synonymous variants (*P* = 0.061, Chi-square test). This result reinforces that genomic context has a direct impact on the likelihood of mutation at a potential hotspot position. Local nucleotide sequence does not operate in isolation but relies on a prominent interplay with genomic position and gene strandedness to drive the specific occurrence of the A289C SNP.

As well as genomic features that are directly involved in the construction of mutational hotspots, it is also important to consider general indirect means by which the mutational spectra can be affected. A prominent example of this is the mismatch repair (MMR) system that is often lacking in mutator strains. MMR systems across numerous bacterial species preferentially correct transition mutations, as mutator strains lacking these genes exhibit transition biases (Schaaper and Dunn 1987; Long et al. 2014). The mutation generated by the *ntrB* 289 hotspot is a transversion mutation, and as such may be more able to dominate the mutational spectrum as the MMR system actively prevents adaptive transitional changes from becoming immortalized in the daughter DNA strands. To test this hypothesis, we constructed and evolved lines of a mismatch defective mutant of AR2 (AR2 Δ*mutS*), which lacks a key part of the MMR protein MutS, the component responsible for binding DNA (Schofield et al. 2001).

We observed that AR2 *ΔmutS* strains displayed a nonsignificant reduced mean time to motility (fig. 3), from 4.20 days in AR2 lines to 2.45 days in the mutator lines (*P* = 0.034, Dunn test). In contrast, the degree of mutational parallelism and spectra across strain backgrounds differed significantly (*P* = 0.0011, Chi-square test). 35% AR2 Δ*mutS* lines fixed *ntrB* A289C, 40% reported SNPs either elsewhere in *ntrB* or in *glnK*, and 25% harbored unidentified mutations outside of these loci. Mutational repeatability of the *ntrB* A289C mutational hotspot had therefore fallen from 95% to 35% in mutator lines. However, this was not owed to a reduction in mutation bias operating at the hotspot, but rather an elevation in the realization of alternative adaptive mutations. *ntrB* A289C mutations were realized sooner in mutator strains (*P* = 0.0012, Wilcoxon test; fig. 3) but so too were alternative transition mutations (identified mutations are plotted in fig 3). The 65% non-A289C mutations in AR2 *ΔmutS* were realized ≤3 days, whereas the 4.8% non-A289C mutations in AR2 were realized ≤6 days. Furthermore, all identified non-A289C mutations in AR2 *ΔmutS* (40% of total sample) were transition mutations.

**Fig. 3.**
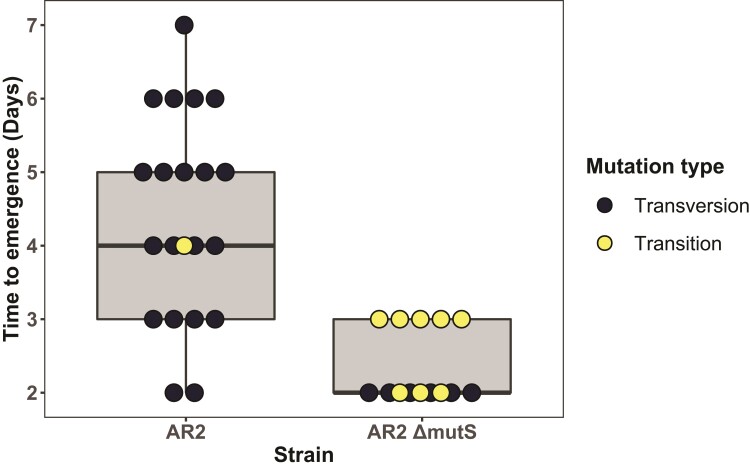
Removal of a mismatch repair complex uncovers alternative adaptive transition mutations. Independent replicates of mutator variants (AR2 Δ*mutS*) realized motility in nonsignificantly less time than the AR2 ancestor (*P* = 0.034, Dunn test) but yielded a significantly different mutational spectra (*P* = 0.0011, Chi-square test). The transversion mutation *ntrB* A289C was observed in 20/21 cases in the ancestral line, with the remaining observation a transition mutation *ntrB* T326C. 15/20 mutator lines had identifiable mutations in *ntrB* or *glnK*; these data points are plotted. 7/15 mutator lines harbored the transversion mutation *ntrB* A289C. The remaining 8/15 were transition mutations: *ntrB* T323C, T407C, A608G (x2), A683G, and *glnK* T11C, A131G, A263G.

A single nucleotide can mutate to three alternate nucleotides, two of which are transversion mutations (e.g., A → C and A → T) and the other a transition mutation (e.g., A → G). Therefore, if we expect transitions to represent 33% of all mutations and assume an equal likelihood of fixation regardless of mutation type, then there is no significant enrichment for either mutation type (transition or transversion) in the mutator lines (Bootstrap test, *n* = 1 × 10^6^, *P* > 0.33). In contrast, there is a significant omission of transitions in an AR2 background where the hotspot transversion remains in effect (Bootstrap test, *n* = 1 × 10^6^, *P* < 0.0023). As such these results show that the mutator strains unlock alternative transition mutations that are suppressed in lines with intact MMR machinery. As the A289C transversion similarly appears more frequently in mutator lines, MMR complexes likely also correct transversion mutations at the hotspot site. Therefore, while the *ntrB* 289 hotspot remains potent in both mutator and nonmutator genomic backgrounds, the mutational spectrum will less heavily favor the hotspot transversion mutation in mutator lines, where alternative mutation types become more common.

Together, these results reveal that genomic position, gene strandedness, local genetic sequence, and the presence of MMR proteins all operate in concert to generate a near-deterministic mutational hotspot. The interplay of these features may be owed to hairpin formation that stalls the replisome at its position, enriching a collision point for the replisome and RNA polymerase. The bias in mutation spectra is additionally indirectly enforced by MMR proteins, which correct alternate adaptive transition mutations. Mutational hotspots have been argued in some cases to be maintained by natural selection, primarily in evolutionary circumstances where a transient and reversible change in phenotype is beneficial (Moxon et al. 2006). However, the biased mutational event occurring via the mechanisms implicated here is likely not reversible, and therefore the hotspot would degrade under fluctuating selection. Instead, it may well be that the genomic context facilitating the hotspot evolved through neutral evolution, which generated the potential for a skewed adaptive landscape (Tenaillon and Matic 2020). If this is the case, it suggests that hotspots may be found throughout bacterial genomes and not merely within alleles under transient selection.

This work helps expand our knowledge of near-deterministic mutational hotspots away from isolated genomic contexts by highlighting interacting mutable genomic features that are each pervasive throughout the bacterial kingdom. As such, future work that quantifies the impact of these features in model organisms other than *P. fluorescens* will help provide a generalizable genomic framework for searching and identifying similarly potent mutational hotspots throughout bacterial genomes. Therefore, this work will aid in facilitating future accurate forecasts of bacterial evolution and contribute toward our understanding of the role that mutation bias plays in determining adaptive evolutionary outcomes.

## Supplementary Material

Supplementary data are available at *Molecular Biology and Evolution* online.

## Supplementary Material

msac132_Supplementary_Data

## Data Availability

All raw data for this study is avaiIable on the Open Science Framework (OSF), and can be accessed at https://osf.io/twcvd/.
