# Supplementary material for: A Near-Deterministic Mutational Hotspot in Pseudomonas fluorescens Is Constructed by Multiple Interacting Genomic Features
Source: Mol Biol Evol. 2022 Jun 16;39(6):msac132. doi: 10.1093/molbev/msac132 (PMC9234803; doi:10.1093/molbev/msac132)
Supplement: msac132_Supplementary_Data [file msac132_supplementary_data.pdf]

Supporting information

Materials and Methods

Strains and culture conditions

All strains were derived from *Pseudomonas fluorescens* AR2 (SBW25Δ*fleQ* IS-ΩKm-hah: PFLU2552), which lacks the flagellum master regulator FleQ and with a transposon-insertional disruption of *viscB* (PFLU2552), rendering it immotile as detailed previously (Alsohim et al. 2014; Taylor et al. 2015). All strains were grown on lysogeny broth (LB; Miller) media at 27°C with 180 r.p.m. shaking. *Escherichia coli* strains were cultured at 37°C. Media supplements are detailed in Supplementary Table S2.

Strain construction by two-step allelic exchange and miniTn7 transposon insertion

Experimental manipulation of the chromosomal location and gene strandedness of *ntrBC* was conducted in an AR2Δ*ntrBC* background generated by two-step allelic exchange following the protocol of Hmelo et al., 2015 with some alterations. In brief, 400bp sequences flanking either side of *ntrBC* were amplified and joined by strand overlap extension (SOE) PCR. This was inserted into allelic-exchange suicide vector pTS1 by SOE-cloning (Bryksin and Matsumura 2010) and transformed into *E. coli* ST18 by chemical-competence heat-shock. *E. coli* ST18 pTS1-Δ*ntrBC* was used in two-parent puddle-mating to transfer the plasmid to AR2. Merodiploids were selected on LB supplemented with Kanamycin sulphate and Tetracycline hydrochloride but lacking 5-ALA supplement required for *E. coli* ST18 growth. Merodiploids were cultured overnight without tetracycline, and plated at a 10^{-5} dilution onto NSLB +15% w/v sucrose agar. Sucrose resistant colonies were isolated and screened for tetracycline sensitivity, and chromosomal presence of the Δ*ntrBC* allele and absence of *ntrBC* coding sequences was confirmed by colony PCR.

Chromosomal reintroduction of *ntrBC* alleles was performed using the miniTn7 transposon insertion system closely following the protocol of Choi & Schweizer, 2006. This reliably introduces miniTn7 downstream *glmS* in *Pseudomonads* without measurable fitness defects (PFLU6114 in *P. fluorescens* SBW25). The entire ntrBC operon, 315bp upstream and 167bp downstream were amplified by colony PCR and transferred to the pJM220 miniTn7 vector (obtained from the Addgene plasmid repository, plasmid number #110559), after removal of

the rhaSR and prhaBAD construct by restriction-ligation cloning. This allowed the native operon structure, promoter, enhancer and terminator of ntrBC to be maintained to avoid alterations to native co-regulation and co-transcription of these genes. Inserts were generated for *ntrBC*, and *ntrBC-sm* which includes the DNA-hairpin abolishing *ntrB* synonymous mutations detailed previously (Horton et al. 2021). These plasmids were transformed into *E. coli* DH5α by chemical-competence heat-shock. Transposon insertion was conducted by four-parent puddle-mating of *E. coli* DH5α miniTn7 plasmid-containing donors with recipient AR2Δ*ntrBC*, and helper strains *E. coli* SM10 λpir pTNS2 and *E. coli* SP50 pRK2073. Transposon insertions were selected for on LB supplemented with Gentamicin sulphate and Kanamycin sulphate. Chromosomal Transposon insertion downstream of *glmS* was confirmed by colony PCR.

Manipulation of *ntrBC* and *ntrBC*-sm gene strandedness was conducted by taking advantage of the reliable insertional orientation of miniTn7 in the SBW25 chromosome where the Tn7R site is always proximal to the 3' end of the *glmS* gene (Choi and Schweizer 2006; Liu et al. 2014). Swapping the positions of the two restriction sites used for cloning the *ntrBC* insert into the miniTn7 transposon plasmid allowed the *ntrBC* inserts to be situated on the opposite strand in relation to Tn7R, and therefore in relation to *glmS*. This allowed reliable insertion of the ntrBC open reading frame (ORF) onto the opposite strand downstream *glmS* (Fig.1A). In this new orientation, the Tn7R site is situated upstream of *ntrBC* genes, so that they are on the same strand as *glmS*, which is the leading strand with respect to the origin of replication. Strand orientation of the *ntrBC* ORF sequence relative to the *glmS* ORF was confirmed by PCR (Supplementary Fig.S2). Ancestral strains were checked for mutations introduced during construction by whole genome resequencing (detailed below). AR2 miniTn7[ntrBC-Lead] was found to have gained a SNP in the *ntrC* gene resulting in the amino acid change D468N. This change was outside of any functional protein motif and had no significant effect on motility phenotype for subsequent mutations (Supplementary Fig.S3).

Motility evolution experiments

AR2 and AR2 miniTn7[*ntrBC*] variants were challenged to rescue motility in the absence of the FleQ master flagellar regulator on soft agar, as described previously (Taylor et al. 2015; Horton et al. 2021). Pure colonies were inoculated into 0.25% agar LB plates as described in Alsohim et al., 2014, and incubated at 27°C. At least 20 replicates were performed for each condition. Plates were checked a minimum of twice daily for motility, recording time to

emergence. The leading edge of motile zones was sampled immediately after emergence by streaking onto LB agar. Single pure colony were re-streaked, and stored at -80°C as glycerol stocks of LB overnight cultures. All subsequent analysis was conducted on these pure motile isolates. Experiment was run for six weeks and any replicates without motility after this cut-off recorded as having not evolved.

Colony PCR and Sanger sequencing to identify mutations

Motility-conferring mutations were identified by colony PCR and subsequent Sanger sequencing (provided by Eurofins Genomics). PCR amplicons were purified using the Monarch® PCR & DNA Cleanup Kit (New England Biolabs). The genes *ntrB, glnK, glnA,* and PFLU1131 were screened, which were selected based on being mutational targets previously known to rescue motility in the AR2 background (Taylor et al. 2015; Horton et al. 2021; Shepherd et al. Unpublished Data). Returned sequences were aligned against the *P. fluorescens* SBW25 reference genome (Silby et al. 2009) using NCBI BLAST to identify mutations. All motile isolates were checked for presence or absence of *ntrB* mutation, and those without an *ntrB* mutation were checked for *glnK, glnA* and PFLU1131 mutation in that order until a mutation was identified.

Whole genome resequencing and calling of SNPs/Indels

Genomic DNA was extracted using the ThermoScientific GeneJET Genomic DNA Purification Kit from ancestral strains, and for motile strains for which no mutation could be identified by Sanger sequencing. Purified gDNA was quality checked using BR dsDNA Qubit spectrophotometry and nanodrop spectrophotometry. Illumina NextSeq 2000 sequencing was provided by the Microbial Genome Sequencing Center (MIGS, Pittsburgh USA), with a minimum 30x coverage. Returned paired-end reads were aligned to the *P. fluorescens* SBW25 reference genome (Silby et al. 2009) using the Galaxy platform (Afgan et al. 2018). Indels were identified using the integrated genomics viewer (Robinson et al. 2011). For SNP identification, the variant calling software SNIPPY was used with default parameters (Seemann, 2015).

Phenotypic assays and analysis

Motility phenotype of AR2 miniTn7[*ntrB'C*] strains measured as distance moved after 24 h of incubation in 0.25% agar LB plates. Six biological replicates were grown as separate overnight cultures. OD_{595} was adjusted so that soft agar plates were inoculated with 1μL of OD_{595} =1

suspension of cells in PBS. The surface of the agar was pierced with the pipette tip, and sample effused into the gap left by the tip. Photographs were taken of motile zones after 24hrs incubation. Surface area moved was calculated from the radius of the concentric motile zone measured from these images ($A = \pi r^2$). Values were square root transformed before plotting.

Growth phenotype in shaking LB broth was measured by inoculating 99 µL of sterile LB broth with 1 µL of the OD_{595} =1 PBS cell suspensions for each replicate in a 96-well plate. Plates were incubated in a plate reader, recording OD_{595} every ten minutes. Area under the bacterial growth curve was calculated as a measure of fitness using the growthcurver package in R and plotted (Sprouffske and Wagner 2016).

RNA extraction, cDNA preparation and RT-qPCR

RNA was extracted from 20 OD units of *P. fluorescens* cultures in mid-log phase growth (OD_{595} ~1.5) in biological triplicates of each strain. At the desired OD, growth and RNA expression was halted by addition of a ½ culture volume of ice-cold killing buffer (20 mM NaN_3, 20 mM Tris-HCl, 5 mM $MgCl_2$). Cells were pelleted, and the killer buffer removed. Pellets were resuspended in buffer RLT from the Qiagen RNeasy extraction kit with β-mercaptoethanol added, and cells lysed by bead-milling at 4500 r.p.m. for 45 s with lysing matrix B. Lysates were added to Qiagen RNeasy extraction columns, and the extraction completed following the kit protocol. A DNase I treatment step (RNase-free DNase kit - Qiagen) was included between buffer RW1 washes, by adding DNase I directly to the column. RNA was eluted in nuclease-free water, and subsequently treated with TURBO DNase from the Turbo DNA-free kit (Invitrogen) following kit protocol.

Purified RNA concentration was measured by Qubit RNA BR assay (ThermoScientific), RNA quality by nanodrop spectrophotometry, and RNA integrity and agarose gel electrophoresis. Production of cDNA for subsequent qPCR was performed from extracted RNA using the Protoscript-II First strand cDNA synthesis kit (New England Biolabs) with random hexamer priming following kit protocols. RT-qPCR was used to measure gene expression of *ntrB* and *ntrC* by the comparative Ct (ΔΔCt) method with *gyrB* as an endogenous reference. Reaction plates were set up using SYBR green PCR master mix (applied biosystems), with cDNA template preps diluted to 10^{-2} in nuclease-free water.

ΔMutS strain assays

AR2Δ*mutS* strains were assembled via two-step allelic exchange as outlined above. Primers used (Supplementary Table S3) produced a knockout construct that deleted nucleotides 760-2554 in the coding region of *mutS*. Three individual Δ*mutS* mutants were isolated and all three utilised at equal frequencies in subsequent experiments and data pooled for analysis. This mitigated the impact of any secondary mutations occurring after knockout construction during -80 banking and routine culturing. To test for mis-match repair deficiency, a fluctuation assay using rifampicin was performed to estimate relative mutation rates between AR2 *mutS* and ancestral AR2. This approach is outlined in (Vogwill et al. 2014), and guided by parameters outlined by (Foster 2006).

In brief, the pooled AR2 Δ*mutS* lines and three AR2 colonies were used to inoculate pre-cultures in 10mL LB liquid broth until early-mid log phase (OD_{595} = 0.4). Precultures were harvested and corrected to OD_{595} = 1. In a 96-well plate, 1μL of cells was added to 99μL LB broth with ten biological replicates for both AR2Δ*mutS* and AR2. The assay was incubated for 23hrs free from antibiotic selection. The contents of each well were plated onto LB plates supplemented with 30μg/mL rifampicin, incubated, and the number of resistant colonies counted. Mutation rate was estimated from the frequency of spontaneous mutations in *rpoB* conferring rifampicin resistance, as a proxy for rates across the genome (Krašovec et al. 2019).

The rates of mutation were determined using FALCOR (Hall et al. 2009). Mutations per generation of the pooled AR2 Δ*mutS* lines was determined as ~3.08 $x10^{-5}$ (95% confidence interval (CI): ~3.19 $x10^{-5}$, ~2.98 $x10^{-5}$) and the pooled AR2 lines as ~5.05 x 10^{-8} (95% CI: ~6.43 $x10^{-8}$, ~3.80 $x10^{-8}$), yielding an approximate 610-fold elevation of mutation rate in the mutator strain. Motility evolution was assayed for the mutator strains and genotypes screened as outlined above.

Statistical analyses

All statistical analysis and data handling was performed using R core statistical packages aside from the Dunn.test package. Shapiro-Wilks normality tests were performed to confirm non-normality of datasets. To compare distributions across two groups, a Wilcoxon rank sum test with continuity correction was performed, with a $P \leq 0.05$ taken to indicate significance. To compare group medians for more than two groups, a Kruskal-Wallis test with post-hoc Dunn test and Benjamini-Hochberg correction was performed, with a $P \leq 0.025$ taken to indicate

significance. To compare frequency counts containing low values, a Pearson's Chi-squared test was used with a Monte Carlo simulation (replicates = 20000) to generate p-values, with $P \leq 0.05$ taken to indicate significance. To compare observed counts against those expected at given probabilities, a Bootstrap test was performed by drawing a subset of values at defined likelihoods 20 times for *n* iterations to form an expected distribution.

Data availability

The data underlying this article are available in the Open Science Framework, at https://dx.doi.org/10.17605/OSF.IO/TWCVD.

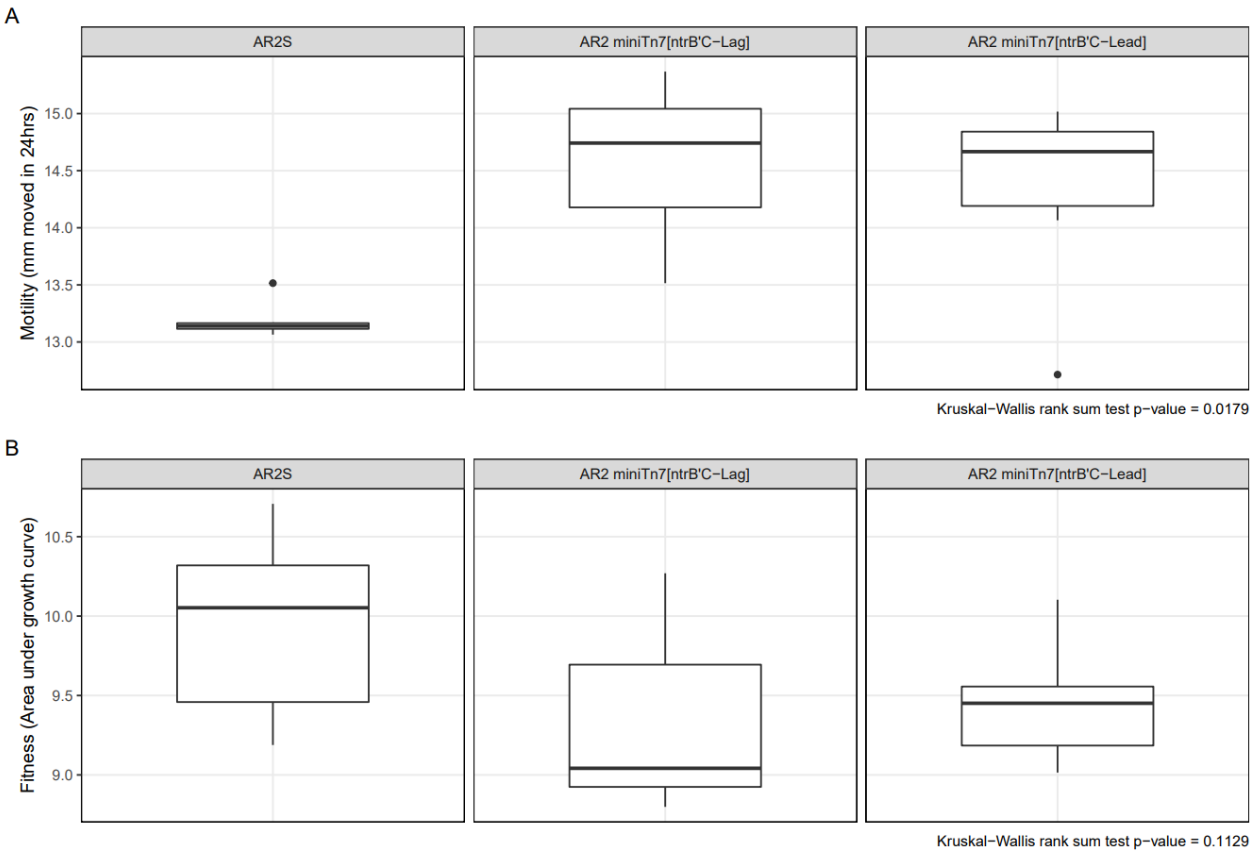

Supplementary Figure S1: Impact of translocation and gene strandedness on motility and shaking LB growth fitness of the *ntrB* A289C mutant. A) Motility of A289C *ntrB* mutation in each genomic topology condition as measured by distance moved after 24 h in LB 0.25% agar (mm). AR2S contains AR2 *ntrB'* (*ntrB* A289C) in its native genomic position and strand orientation. No significant difference was found between AR2 miniTn7[*ntrB'C*-Lag] and AR2 miniTn7[*ntrB'C*-Lead] (P= 0.2667, Dunn test), however both moved significantly further than AR2S (*P*= 0.0034 and *P*= 0.0185 respectively, Dunn test). **B)** Fitness of A289C *ntrB* mutation for each genomic topology condition as measured by area under the growth curve for 24 h growth in shaking LB broth. No significant differences were found between any strain tested (*P*= 0.1129, Kruskal-Wallis test).

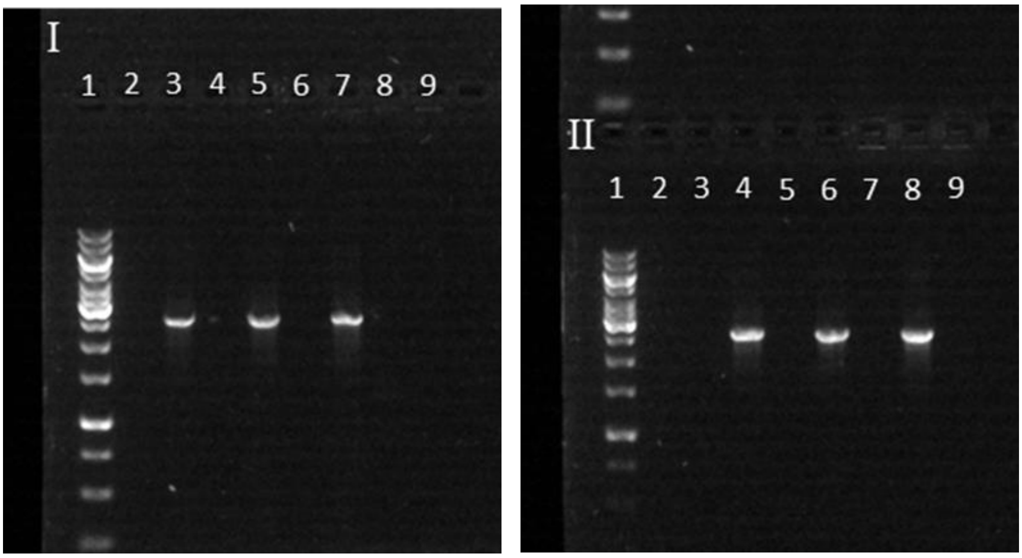

Supplementary Figure S2: **Agarose gel demonstrating difference in strand orientation between miniTn7[*ntrBC*-Lag] and miniTn7[*ntrBC*-Lead] engineered strains.** Primer pair 'LAG' (SBW25-glmS + ntrC-Down-F) will only amplify if *ntrBC* are downstream of *glmS* and coding on the lagging strand. Primer pair 'LEAD' (SBW25-glmS + ntrBC-KO-up-R) will only amplify if *ntrBC* are downstream of *glmS* and coding on the leading strand (see Supplementary Table S3 for primer information). Well contents: **Row 'I':** 1 – 1Kb Generuler DNA ladder. 2 – LEAD negative control. 3 – LAG AR2 miniTn7[*ntrBC*-Lag]. 4 – LEAD AR2 miniTn7[*ntrBC*-Lag]. 5 – LAG AR2 miniTn7[ntrBC-sm-Lag]. 6 – LEAD AR2miniTn7[*ntrBC*-sm-Lag]. 7 - LAG AR2 miniTn7[*ntrB'C*-Lag]. 8 – LEAD AR2miniTn7[*ntrB'C*-Lag]. 9 – LEAD AR2. **Row 'II':** 1 – 1Kb Generuler DNA ladder. 2 – LAG negative control. 3 – LAG AR2 miniTn7[*ntrBC*-Lead]. 4 – LEAD AR2 miniTn7[*ntrBC*-Lead]. 5 – LAG AR2 miniTn7[*ntrBC*-sm-Lead]. 6 – LEAD AR2 miniTn7[*ntrBC*-sm-Lead]. 7 - LAG AR2miniTn7[*ntrB'C*-Lead]. 8 – LEAD AR2 miniTn7[*ntrB'C*-Lead]. 9 – LAG AR2.

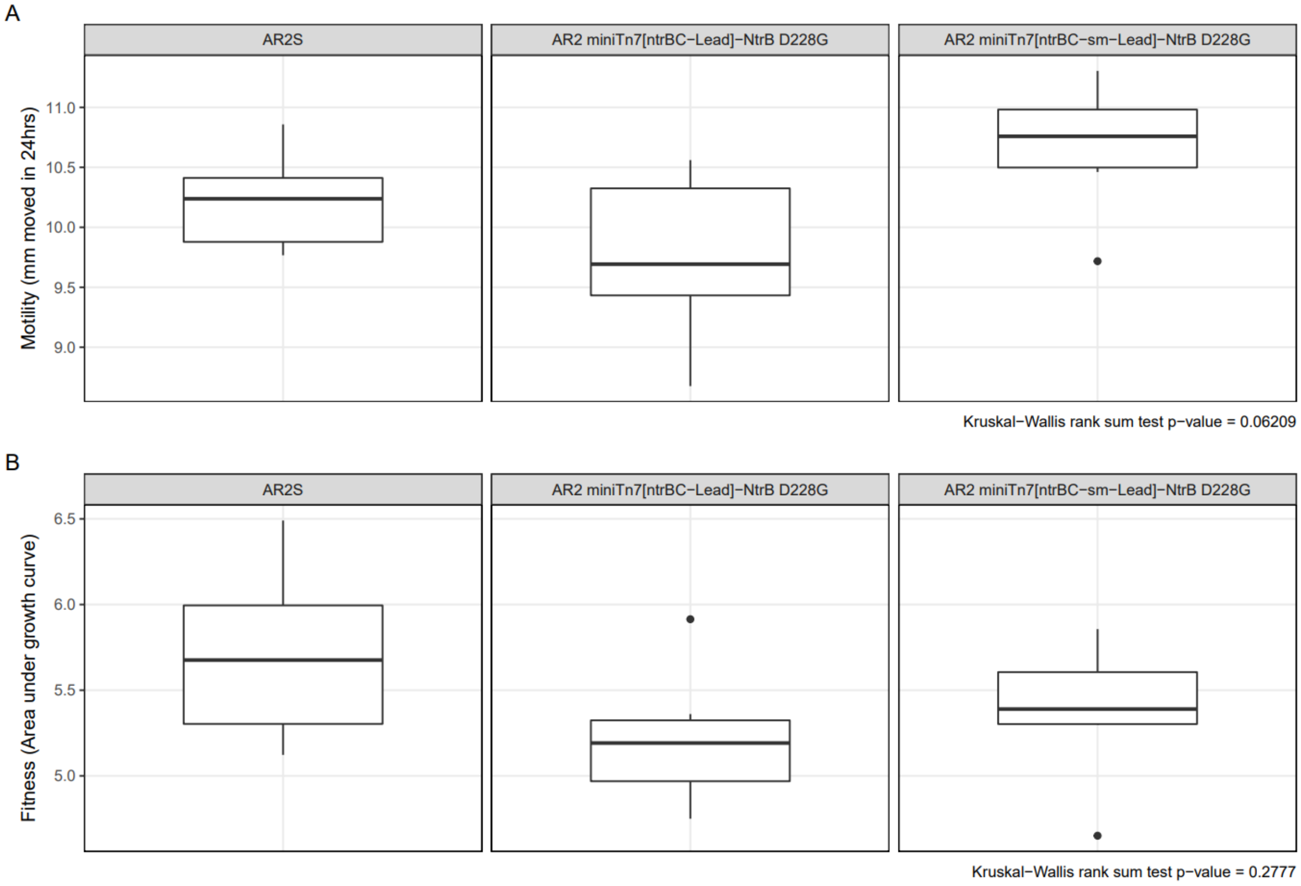

Supplementary Figure S3: NtrC D468N mutation introduced during cloning has no impact on subsequent motility phenotypes. AR2 miniTn7[ntrBC-Lead] with the NtrB mutation D228G (AR2 miniTn7[ntrBC-Lead] strain S-1) and the illicit NtrC D468N is compared with an AR2 miniTn7[ntrBC-sm-Lead] strain with the same NtrB D228G mutation but lacking NtrC D468N (AR2 miniTn7[ntrBC-sm-Lead] strain S-25). No significant difference was found between the two strains for motility in soft agar (**A**) and growth in shaking LB broth (**B**) assays (*P*= 0.06209 and *P*= 0.2777 respectively, Kruskal-Wallis tests).

Supplementary Table S1: No significant changes in expression of the *ntrB* or *ntrC* genes was observed for each genomic topology condition. Expression was assayed for biological triplicates each in technical triplicates, using the comparative Ct (ΔΔCt) method with *gyrB* as an endogenous reference. P-values produced using Dunn tests.

Strain	*ntrB*	
	Fold change relative to AR2	p-value
AR2 miniTn7[*ntrBC* -Lag]	0.774836401	0.4543
AR2 miniTn7[*ntrBC* -Lead]	1.314879894	0.2036
	ntrC	
	Fold change relative to AR2	p-value
AR2 miniTn7[*ntrBC* -Lag]	1.095604638	0.4226
AR2 miniTn7[*ntrBC* -Lead]	1.784101725	0.2189

Supplementary Table S2: Pair-wise comparisons of mutation spectra at locus level between strains broadly support a replisome-RNA polymerase collision hypothesis, as denoted by the key. P-values produced using a Pearson's Chi-squared test with a Monte Carlo simulation (replicates = 20000).

Strain<br>AR2 miniTN7[*ntrBC*-]	sm-Lag	Lead	sm-Lead
Lag	0.0074	0.00005*	0.00005*
sm-Lag	x	0.19*	0.011*
Lead	x	x	0.51#
sm-Lead	x	x	x

Key:
P value < 0.05
* Significant difference expected
# Significant difference not expected

Supplementary Table S3: Bacterial strains and culture conditions used in this study

Strain	Media supplement
P. fluorescens AR2 and derivatives	Kanamycin sulphate 50µg/mL
P. fluorescens AR2 derivatives containing miniTn7 transposons	Kanamycin sulphate 50µg/mL, Gentamicin sulphate 5µg/mL
E. coli DH5α containing pJM220-derived plasmids	Ampicillin sodium salt 100µg/mL,<br><br>Gentamicin sulphate 5µg/mL
E. coli SM10 λpir pTNS2	Ampicillin sodium salt 100µg/mL
E. coli SP50 pRK2073	Streptomycin sulphate 100µg/mL
E. coli ST18 derived strains	5-aminolevulinic acid hydrochloride (5-ALA) 50µg/mL
E. coli containing pTS1-derived plasmids, and *P. fluorescens* allelic exchange merodiploids	Tetracycline hydrochloride 10µg/mL

Supplementary Table S4: Details of oligonucleotide primers used in this work.

Primer name	Sequence 5'-3'	Purpose
ntrC-down-R	GAAATTAATAGGTTGTATTGATGTTGTACCAGGGCTCCCAAAAC	Amplify downstream homologous region of ntrBC for knockout allelic exchange. Introduces overlap for incorporation into pTS1 vector by SOE-cloning.
ntrBC-KO-down F	GACCATCAGCGATGCACTGGGCGATGAAGGCTGAATC	
ntrBC-KO-up-R	GATTCAGCCTTCATCGCCCAGTGCATCGCTGATGGTC	Amplify upstream homologous region of ntrBC for knockout allelic exchange. Introduces overlap for incorporation into pTS1 vector by SOE-cloning.
ntrBC-KO-up-F	GCCGTTTCTGTAATGAAGGAGAAAACCCGAGATGGTAGGCATTGAAC	
mutS_out_NF	GCATGGGCGACTTCTACGAG	Amplify upstream homologous region of mutS for knockout allelic exchange. Contains complementary overhangs with downstream fragment.
mutS_ins_NR	GTGCATATAACATTTCGAGCGCAGGGCGGTGCGCTGGGTTT	
mutS_ins_CF	AAACCCAGCGCACCGCCCTGCGCTCGAAATGTTATATGCAC	Amplify downstream homologous region of mutS for knockout allelic exchange. Contains complementary overhangs with upstream fragment.
mutS_out_CR	AATTTAAGCTTCGCTATCAGCGTTCGAGGTC	
mutS_nest_NF	AATTTGGATCCGTTGCTGGACATCACCCTG	Amplifies across annealed upstream and downstream mutS fragments for knockout allelic exchange. Introduces restriction enzyme recognitions sites BamH1 and HindIII to termini of fragment for incorporation into pTS1 vector by restriction-ligation.
mutS_nest_CR	AATTTAAGCTTACTTCGTCCTCGGAGAAAAT	
ntrBC-SacI-F	AATTTGAGCTCCACTGTCCGAACAACACTGATC	Amplify ntrBC and their promoter+terminator regions, introducing a SacI site upstream, and a HindIII site downstream.
ntrBC-HindIII-R	AATTTAAGCTTCGGTTCATGGTGCATTGAAGC	
ntrBC-HindIII-F	AATTTAAGCTTCACTGTCCGAACAACACTGATC	Amplify ntrBC and their promoter+terminator regions, introducing a HindIII site upstream, and a SacI site downstream.
ntrBC-SacI-R	AATTTGAGCTCCGGTTCATGGTGCATTGAAGC	
SBW25-glmS	CACCAAAGCTTTCACCACCCAA	SBW25 glmS primer sequence from Liu et al., 2014.<br><br>Pair checks insertion of ntrBC for lagging strand orientation.
ntrC-Down-F	GACATGAGCCGTAGTGAAACCGGGCGATGAAGGCTGAATC	
ntrB-1119-F	GAGGTCCCAATGACCATCAG	Amplification of ntrB for Sanger sequencing.
ntrB-1119-R	GACGATCCAGACGGTTTCAC	
glnK-F	GTGGGCAAAGGACTGATTTC	

glnK-R	GATGATGGCGAAGGTCATCT	Amplification of glnK for Sanger sequencing.
PFLU1131-F	CGATAAGCGAAACCTGATG	Amplification of PFLU1131 for Sanger sequencing.
PFLU1131-R	CGACTACCAGAATGTTATGCG	
glnA-F	CGGAAATCGCTCAAGGTTTA	Amplification of glnA for Sanger sequencing.
glnA-R	CTGATAATCCCCAGGCAAAA	
glnK-long-F	CTCCAGGTTCTCCAGGCG	Amplify the glnK and amtB locus for Sanger sequencing of larger deletion mutants.
glnK-long-R	GCCCATCGGCGCGCATTC	
ntrB-F-qPCR	CTTGCGCCTTGAGTACATGA	RT-qPCR primer pair for ntrB – from Taylor et al., 2015.
ntrB-R-qPCR	GTTGCTCAGGATAGGGGTC	
ntrC-F-qPCR	GCCGTAGTGAAACCGTCTG	RT-qPCR primer pair for ntrC– from Taylor et al., 2015.
ntrC-R-qPCR	CATGCGGATGTCGGAGATG	
gyrB-F-qPCR	CGTCACACCATCCAGCGAT	RT-qPCR primer pair for gyrB– from Taylor et al., 2015.
gyrB-R-qPCR	AAGTCACGACGAGGCTCGA	

Supplementary References

Afgan E, Baker D, Batut B, Van Den Beek M, Bouvier D, Ech M, Chilton J, Clements D, Coraor N, Grüning BA, et al. 2018. The Galaxy platform for accessible, reproducible and collaborative biomedical analyses: 2018 update. *Nucleic Acids Res.* 46:W537–W544.

Alsohim AS, Taylor TB, Barrett GA, Gallie J, Zhang X, Altamirano-Junqueira AE, Johnson LJ, Rainey PB, Jackson RW. 2014. The biosurfactant viscosin produced by Pseudomonas fluorescens SBW25 aids spreading motility and plant growth promotion. *Environ. Microbiol.* 16:2267–2281.

Bryksin A V., Matsumura I. 2010. Overlap extension PCR cloning: a simple and reliable way to create recombinant plasmids. *Biotechniques* 48:463–465.

Choi KH, Schweizer HP. 2006. mini-Tn7 insertion in bacteria with single attTn7 sites: Example Pseudomonas aeruginosa. *Nat. Protoc.* 1:153–161.

Foster PL. 2006. Methods for Determining Spontaneous Mutation Rates. *Methods Enzym.* 409:195–213.

Hall BM, Ma C, Liang P, Singh KK. 2009. Fluctuation AnaLysis CalculatOR: a web tool for the determination of mutation rate using Luria–Delbrück fluctuation analysis. *Bioinformatics* 25:1564–1565.

Hmelo LR, Borlee BR, Almblad H, Love ME, Randall TE, Tseng BS, Lin C, Irie Y, Storek KM, Yang JJ, et al. 2015. Precision-engineering the Pseudomonas aeruginosa genome with two-step allelic exchange. *Nat. Protoc.* 10:1820–1841.

Horton JS, Flanagan LM, Jackson RW, Priest NK, Taylor TB. 2021. A mutational hotspot that determines highly repeatable evolution can be built and broken by silent genetic changes. *Nat. Commun.* 12:1–10.

Krašovec R, Richards H, Gomez G, Gifford DR, Mazoyer A, Knight CG. 2019. Measuring microbial mutation rates with the fluctuation assay. *J. Vis. Exp.* 2019:1–9.

Liu Y, Rainey PB, Zhang XX. 2014. Mini-Tn7 vectors for studying post-transcriptional gene expression in Pseudomonas. *J. Microbiol. Methods* 107:182.

Robinson JT, Thorvaldsdóttir H, Winckler W, Guttman M, Lander ES, Getz G, Mesirov JP. 2011. Integrative genomics viewer. *Nat. Biotechnol.* 29:24–26.

Silby MW, Cerdeño-Tárraga AM, Vernikos GS, Giddens SR, Jackson RW, Preston GM, Zhang XX, Moon CD, Gehrig SM, Godfrey SAC, et al. 2009. Genomic and genetic analyses of diversity and plant interactions of Pseudomonas fluorescens. *Genome Biol.* 10:1–16.

Sprouffske K, Wagner A. 2016. Growthcurver: An R package for obtaining interpretable metrics from microbial growth curves. *BMC Bioinformatics* 17:17–20.

Taylor TB, Mulley G, Dills AH, Alsohim AS, McGuffin LJ, Studholme DJ, Silby MW, Brockhurst MA, Johnson LJ, Jackson RW. 2015. Evolutionary resurrection of flagellar motility via rewiring of the nitrogen regulation system. *Science (80-.).* 347:1014–1017.

Vogwill T, Kojadinovic M, Furió V, Maclean RC. 2014. Testing the role of genetic background in parallel evolution using the comparative experimental evolution of antibiotic resistance. *Mol. Biol. Evol.* 31:3314–3323.
